# Cluster headache attack remission with sphenopalatine ganglion stimulation: experiences in chronic cluster headache patients through 24 months

**DOI:** 10.1186/s10194-016-0658-1

**Published:** 2016-07-26

**Authors:** Mads C. J. Barloese, Tim P. Jürgens, Arne May, Jose Miguel Lainez, Jean Schoenen, Charly Gaul, Amy M. Goodman, Anthony Caparso, Rigmor Højland Jensen

**Affiliations:** 1 Danish Headache Centre, Department of Neurology, Rigshospitalet-Glostrup, University of Copenhagen, Copenhagen, Denmark; 2 Department of Clinical Physiology, Nuclear Medicine and PET, Rigshospitalet-Glostrup, Copenhagen, Denmark; 3 Department of Neurology, University Medical Center Rostock, Rostock, Germany; 4 Department of Systems Neuroscience, University Medical-Center Hamburg-Eppendorf, Hamburg, Germany; 5 Department of Neurology, Hospital Clinico Universitario, Universidad Católica de Valencia, Valencia, Spain; 6 Headache Research Unit. Department of Neurology - CHR Citadelle, Liège University, Liège, Belgium; 7 Migraine- and Headache Clinic Königstein, Königstein, Germany; 8 Clinical Research, Autonomic Technologies, Redwood City, California, USA

**Keywords:** Cluster headache, Remission, Neuromodulation, Neurostimulation, Sphenopalatine ganglion

## Abstract

**Background:**

Cluster headache (CH) is a debilitating headache disorder with severe consequences for patient quality of life. On-demand neuromodulation targeting the sphenopalatine ganglion (SPG) is effective in treating the acute pain and a subgroup of patients experience a decreased frequency of CH attacks.

**Methods:**

We monitored self-reported attack frequency, headache disability, and medication intake in 33 patients with medically refractory, chronic CH (CCH) in an open label follow-up study of the original Pathway CH-1 study. Patients were followed for at least 24 months (average 750 ± 34 days, range 699-847) after insertion of an SPG microstimulator. Remission periods (attack-free periods exceeding one month, per the ICHD 3 (beta) definition) occurring during the 24-month study period were characterized. Attack frequency, acute effectiveness, medication usage, and questionnaire data were collected at regular clinic visits. The time point “after remission” was defined as the first visit after the end of the remission period.

**Results:**

Thirty percent (10/33) of enrolled patients experienced at least one period of complete attack remission. All remission periods followed the start of SPG stimulation, with the first period beginning 134 ± 86 (range 21-272) days after initiation of stimulation. On average, each patient’s longest remission period lasted 149 ± 97 (range 62-322) days. The ability to treat acute attacks before and after remission was similar (37 % ± 25 % before, 49 % ± 32 % after; *p* = 0.2188). Post-remission headache disability (HIT-6) was significantly improved versus baseline (67.7 ± 6.0 before, 55.2 ± 11.4 after; *p* = 0.0118). Six of the 10 remission patients experienced clinical improvements in their preventive medication use. At 24 months post insertion headache disability improvements remained and patient satisfaction measures were positive in 100 % (10/10).

**Conclusions:**

In this population of 33 refractory CCH patients, in addition to providing the ability to treat acute attacks, neuromodulation of the SPG induced periods of remission from cluster attacks in a subset of these. Some patients experiencing remission were also able to reduce or stop their preventive medication and remissions were accompanied by an improvement in headache disability.

## Background

Cluster headache (CH) is considered the most painful primary headache disorder. In the episodic form (ECH) bouts of attacks alternate with periods of remission of varying duration. In the chronic form such remissions are lacking or shorter than 1 month through at least 1 year [1]. Because of the multiple daily attacks, social and economic consequences for patients are severe. CCH impairs daily functioning and reduces quality of life: 75 % of patients report severe disability, 20 % have suicidal tendencies, and 20 % are unemployed or receive government disability compensation [2–4]. A study of 179 patients in Germany concluded that mean annual direct and indirect costs were significant [5].

Standard treatment includes verapamil and/or lithium as prophylactic therapy and oxygen or triptans (injectable or nasal) for acute attacks [6]. Still, many CH patients are undertreated due to insufficient management or risk factors and side effects associated with established treatment options. Moreover, a subset of CH patients are refractory or have only limited benefit from standard medical treatments (see [7] for definition of refractory CH). New, promising treatment options have become available that may provide relief to these insufficiently treated patients. Among them, neuromodulation has become a viable clinical option thanks to technological advances.

The prominent autonomic features and the pain during CH attacks are attributed to activation of a trigemino-autonomic reflex that is mediated through the sphenopalatine ganglion (SPG) (also called the pterygopalatine ganglion). The SPG connects directly and indirectly with the hypothalamus, superior salivatory nucleus (SSN), trigemino-vascular system, meninges, and somatic and autonomic nerves innervating cranial structures and is thus a crucial part of the trigemino-parasympathetic circuit and a well-recognized target of therapeutic intervention, including neuromodulation [8, 9]. Various direct manipulations of the SPG including steroid/alcohol injections, radiofrequency ablation, surgical resection, and gamma knife surgery act as proofs of concept but are not viable clinical options, as therapeutic effects may be short-lasting, requiring repeated procedures, or side-effects are poorly tolerated.

The development of an implantable microstimulator affixed to the maxilla, with electrodes placed in the pterygopalatine fossa proximate to the SPG, allows for reversible, repeated, targeted stimulation of the SPG, and its short- and long-term acute effects and safety have already been documented [10–12]. SPG stimulation, with the correct stimulation paradigm, likely interrupts the trigemino-autonomic reflex inhibiting efferent outflow, i.e. the final common pathway for parasympathetic activation in CH [13, 14].

Although not an endpoint of the original, randomized, controlled Pathway CH-1 study, the investigators became aware of an unexpected preventive effect, in addition to the already reported acute effect of SPG stimulation [11]. After stimulation was initiated, a subset of patients began to notice reductions in cluster attack frequency and had attack-free periods, lasting weeks or months. We aimed to characterize these remission periods in more detail over a follow-up period of 24 months.

## Methods

In the original Pathway CH-1 study, CCH patients underwent trans-oral insertion of a microstimulator, such that the stimulating electrodes were placed sufficiently proximate to the SPG to allow for targeted neurostimulation of this structure. Additional insertion details have previously been published [15, 16]. Patients were followed for 12 months following microstimulator insertion. During this time, efficacy of SPG stimulation was initially evaluated in a randomized, controlled, experimental period [11]. Following this phase and during the first 12 months post insertion, patients had the opportunity to use the therapy in an open label period. After the first 12 months an extended long term follow-up study (LTFU) was initiated so that follow-up beyond the original study was possible. Visits during the first 12 months post-insertion occurred at intervals ranging from every two weeks to every three months, and during the second year, every three months. Data from microstimulator insertion through 24 months post-insertion were analysed, and results are presented for patients enrolled in the extended follow-up study who experienced remission from attacks.

### Patient selection

Inclusion and exclusion criteria for the original Pathway CH-1 study and the LTFU have been published previously [11, 12]. Briefly, participants were medically refractory CCH patients with a minimum of four attacks/week [17, 18]. Enrolment in the LTFU required participation in the Pathway CH-1 study, continued microstimulator implantation, compliance with study protocol, and written informed consent. Reasons for not being enrolled in the LTFU included unwillingness to follow the protocol or lack of signed informed consent. Both studies were approved by the appropriate competent national, regional, and/or institutional review boards at all participating centers. Studies were registered on clinicaltrials.gov (NCT01255813 and NCT01616511).

### Data collection

Acute response to SPG stimulation was captured prospectively in an electronic headache diary incorporated into the remote controller. Pain scores are reported using the Categorical Pain Scale (CPS) [0-none, 1-mild, 2-moderate, 3-severe, 4-very severe]. Headache pain was reported prior to stimulation use, upon turning on the remote control, and again along with acute medication use at either 15 min following the start of stimulation (0-12 months post-insertion), or immediately following cessation of each SPG stimulation session (12-24 months post-insertion). Attack frequency (recollection or diary over past four weeks), preventive medications, and headache disability (HIT-6 headache impact test) [19] were captured prior to implant and at each study visit referring to the previous 4 weeks. With regards to the study visits and duration, 28 days was considered to be one month.

### Outcomes and analyses

During the second year of the study, clinic visits occurred every 3 months, and patients were asked to record their average cluster attack frequency over the prior 4 weeks. Attack frequency data were imputed using data from the subsequent clinic visit. Post-hoc analyses evaluating attack frequency over the entire 24 month study period were performed. Per the ICHD-3 (beta) criteria, a cluster remission period is defined as “the time during which attacks cease to occur spontaneously and cannot be induced with alcohol or nitroglycerine. To be considered a remission the attack-free period must exceed one month”[1]. In the analysis herein, remission patients experienced zero cluster attacks for longer than one month following active use of SPG stimulation, as reported on the case report form. Induction of attacks using alcohol or nitroglycerine was not attempted.

Acute effectiveness is defined as pain relief (decrease in CPS score from 2 (moderate) or greater to 1 (mild) or 0 (none) without the use of acute medications) or pain freedom (CPS score decreased from 1 (mild) or greater to 0 (none) without the use of acute medications) following SPG stimulation of an acute attack. Acute effectiveness was evaluated in all patients with remission for all evaluable SPG stimulation attempts (i.e., those with completed electronic headache diary data; sham and sub-perception attempts during the randomized, controlled experimental period were excluded). Acute effectiveness pre- and post-remission was compared using the Wilcoxon signed rank test. In patients experiencing more than one remission period, the longest remission period was used.

Use of preventive medications, headache disability, and patient experiences with SPG stimulation were evaluated at the study visit following the longest remission period at which these data were collected. Within-patient, headache disability improvements were considered clinically significant if scores improved by at least 2.5 units relative to baseline [19]. Differences in baseline characteristics between patients with remission and those without were calculated using *t*-test (age, years of CH, HIT-6), Fisher’s exact test (gender), and Wilcoxon rank sum test (attack frequency).

### SPG Microstimulator system

The SPG Microstimulator (also referred to as the ATI Neurostimulator and the Pulsante^TM^ SPG Microstimulator) is designed to fit the facial anatomy with an integrated lead placed proximate to the SPG. The microstimulator communicates with a handheld remote controller using radiofrequency, is inductively powered, and contains no battery [11]. Using the remote controller, patients apply on-demand SPG stimulation to treat the acute pain of their cluster attacks. Later in the study, some patients started stimulating prophylactically, i.e. in a painless state, without treating acute attacks, as a result of the unanticipated finding of a preventative effect of SPG stimulation. As this was unexpected, no data was systematically collected regarding prophylactic stimulation. Electronic diaries indicate that some patients used prophylactic stimulation extensively, others sparsely.

## Results

Thirty-three medically refractory CCH patients participated in the extended follow-up through 24 months post-insertion (Table 1). Average time from microstimulator insertion through 24 months post-insertion was 750 ± 34 days, (range 699-847). Average duration of CH at baseline was 10.5 ± 8.3 years (range 1-36), with an average attack frequency of 16.8 ± 13.7 attacks/week (range 5-70).

**Table 1:** Clinical baseline characteristics (mean ± SD) from the total population and sub-populations of patients with and without remission are presented

Clinical baseline characteristics	Total *(N = 33)*	CCH with no remission *(N = 23)*	CCH with remission *(N = 10)*	*P*-values (with and without remission)
Age	41.5 ± 12.0	40.7 ± 12.7	43.3 ± 10.7	*p* = 0.5698
Male/female	5.6:1	6.6:1	4:1	*p* = 0.6269
Attacks/week at baseline (recalled over 4 weeks)	16.8 ± 13.7	16.6 ± 14.1	17.3 ± 13.3	*p* = 0.9689
Years of CH at baseline	10.5 ± 8.3	11.0 ± 9.6	9.6 ± 4.6	*p* = 0.5851
HIT-6 score at baseline	66.7 ± 6.2	66.2 ± 6.4	67.7 ± 6.0	*p* = 0.5366

**Fig. 1:**
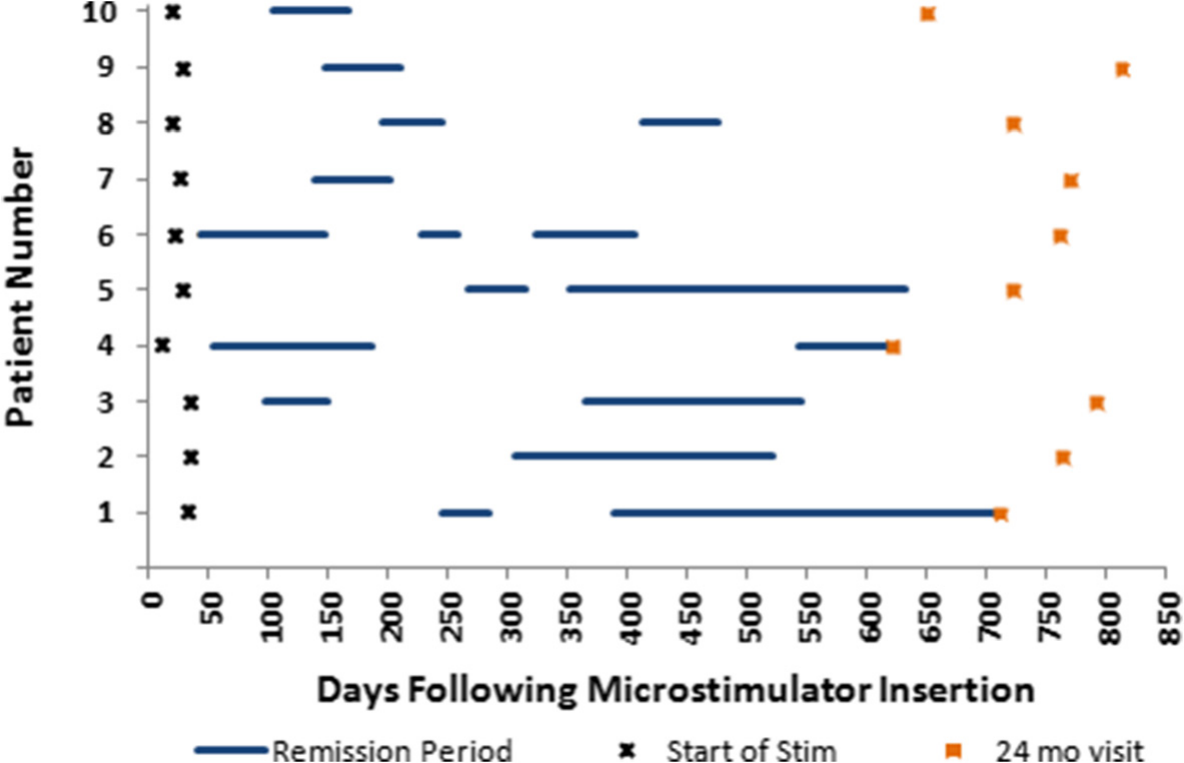
Remission Periods per patient. periods of complete attack remission for the 10 patients experiencing remission. Data through the entire study period, from microstimulator insertion to the 24 month study visit are provided. X’s indicate first use of stimulator. Squares indicate 24 month study visit

Ten patients (30 %) experienced one or more remission periods lasting at least one month (Fig. 1). One additional patient experienced a continuous period of 28 days without attacks following initiation of stimulation, but was not included in the analysis. The initial attack free period occurred after the start of SPG stimulation in all 10 patients: 159 ± 91 days, range 42-306, following microstimulator insertion and 134 ± 86 days, range 21-272, following start of stimulation. For these 10 patients, at the clinic visit when stimulation was first started, average attack frequency was 16.4 ± 16.8 attacks/week (range 3.5-60, compared with baseline (all 33 patients) *p* = 0.7090). Each patient’s longest attack-free period lasted 149 ± 97 days (range 62-322). The number of distinct remission periods varied between patients, with four patients experiencing only one, five patients experiencing two, and one patient experiencing three distinct remission periods. During the remission periods, patients used SPG stimulation as in a painless state (i.e. as prevention), on average, 1.3 ± 1.5 (range 0-4.3) times per week although no specific instructions regarding stimulator use outside of attacks were provided by investigators. Before and after the longest remission period, this patient population (*N* = 10) treated 374 and 217 evaluable attacks, respectively. Average acute effectiveness per patient was unchanged pre- and post-remission (*p* = 0.2188) (Table 2).

**Table 2:** Average acute effectiveness, assessed using the Wilcoxon signed rank test, is unchanged following each patient’s longest cluster attack remission (*p* = 0.2188)

Acute outcomes in patients with remission						
Remission patient	Attack frequency (attacks/wk) at baseline	Days of stimulation prior to first remission	Consecutive (longest) remission duration (days)	Calendar months during (longest) remission	% of attacks achieving effective therapy (# effective therapy/total treated)	
					Before Remission	After Remission
1	45	212	322	Feb -> Nov	0 % (0/203)	0 % (0/1)
2	8	272	215	Dec -> Aug	62 % (18/29)	64 % (32/50)
3	7	63	182	Dec -> Jun	0 % (0/4)	46 % (6/13)
4	35	42	133	Aug -> Dec	33 % (3/9)	7 % (2/30)
5	16	238	280	Jul -> Apr	57 % (12/21)	52 % (12/23)
6	10	21	106	Jul -> Nov	38 % (11/29)	43 % (3/7)
7	20	113	64	Aug -> Oct	21 % (4/19)	56 % (14/25)
8	7	174	63	Mar -> May	47 % (14/30)	100 % (33/33)
9	5	119	63	Oct -> Dec	36 % (4/11)	33 % (1/3)
10	20	84	62	Apr -> Jun	74 % (14/19)	91 % (29/32)
Avg ± SD	17.3 ± 13.3	133.8 ± 86.3	149.0 ± 96.7	-	36.8 ± 31.8 %	49.2 ± 31.8 %
					(*p* = 0.2188)	

### Improvements in headache impairment and acute and preventive medications post-remission

**Fig. 2:**
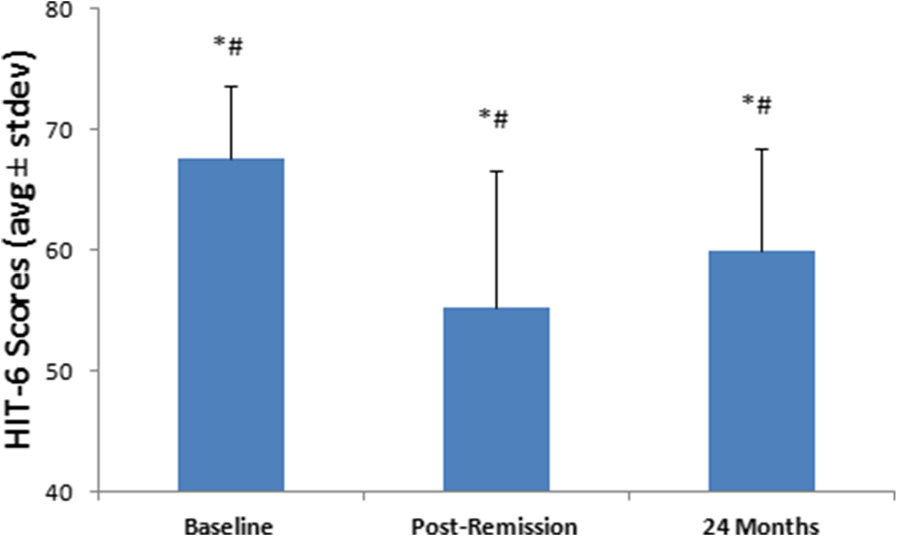
HIT-6 Headache Disability Changes. HIT-6 scores in patients with remission (*N* = 10). Improvements in HIT-6 scores from baseline in these 10 patients are clinically (*) and statistically (#) significant at both the post-remission and 24 month evaluation points

Post-remission headache impairment and medication data collection coincided with the visit at which the longest remission period ended for 7 patients, occurred at a clinic visit following the longest remission in 2 patients, and occurred at a clinic visit after the start of, but prior to the ending of the longest remission period in 1 patient. The 10 patients experiencing cluster attack remission were all severely disabled by their headaches prior to entering the Pathway CH-1 study, scoring 67.7 ± 6.0 (range 58-76) on the HIT-6. Following the remission, scores improved to 55.2 ± 11.4 (range 40-73) (Fig. 2). The improvement was more than eight times the between-group minimally important difference of -1.5 [19] and was statistically significant (*p* = 0.0118). Patients experiencing remission during the first 24 months of microstimulator use continued to see improvements in headache impairment; 70 % (7/10) were HIT-6 responders, experiencing a clinically meaningful improvement in headache disability. Impairment scores in patients experiencing remission (67.7 ± 6.0 at baseline improved to 60.0 ± 8.5 at 24 months) were not different from non-remission patients’ (66.2 ± 6.4 at baseline improved to 62.4 ± 9.7 at 24 months) (*p* = 0.1997).

All ten patients who experienced remission used triptans at baseline. At 24 months post-insertion, 6/10 used no triptans, with 3/10 using no acute treatments at all (Table 3). Clinical improvements in preventive CH medication use (reduction in dose, stopping medications, or remaining off medications) were observed in 4/10 patients post-remission, and 6/10 patients at 24 months post-insertion (Table 3). There were no obvious patterns in dose changes before or during remission periods.

**Table 3:** Post-remission indicates the first visit after the remission period ended where attack frequency, medication, and questionnaire data were collected; In two patients (patients 1 and 10), the visit at which medication data were collected (i.e., post-remission visit) occurred after remission had started, and the remission period had not yet ended at that time

Acute and preventive medication use in patients with remission						
Remission patient	Acute CH medications			Preventive CH medications		
	Baseline	Post-remission	24 Month	Baseline	Post-remission	24 Month
1	Zolmitriptan	Oxygen*	Oxygen*	Valproic acid	None*	None*
2	Sumatriptan, oxygen	None*	Sumatriptan, oxygen	Verapamil	Verapamil	Verapamil, gabapentin
3	Sumatriptan	Sumatriptan	Sumatriptan	Verapamil, lithium	Verapamil, lithium	None*
4	Sumatriptan	None*	None*	Topiramate	None*	None*
5	Sumatriptan	None*	None*	Verapamil, lithium, gabapentin	Gabapentin	Gabapentin*
6	Zolmitriptan, oxygen	Frovatriptan	Oxygen*	Verapamil, lithium	None*	Verapamil
7	Sumatriptan, oxygen	None*	Sumatriptan	Verapamil, lithium, gabapentin	Verapamil, lithium, gabapentin	Verapamil, gabapentin, lithium
8	Sumatriptan, oxygen	None*	None*	Candesartan	Candesartan	Candesartan
9	Sumatriptan	n.a.	Sumatriptan	None	n.a.	None*
10	Sumatriptan, oxygen	None*	Oxygen*	Verapamil	None*	None*

Data on post-remission acute and preventive CH medications are not available in patient 9 (n.a.). *Patients who stopped acute triptan use or had a clinical improvement in or remained off all preventive meds

All 10 remission patients also completed a questionnaire evaluating their experience with SPG stimulation at 24 months. All indicated that SPG stimulation was useful for treating their headaches, and they would recommend SPG stimulation to someone else suffering from CH. All 10 also indicated they would make the same decision again to use SPG stimulation to treat their attacks, and that they found the inserted microstimulator comfortable and the sensation of stimulation tolerable.

## Discussion

In addition to the acute and preventive effects previously reported [11, 12], long-term SPG stimulation induced a state of headache remission of varying duration in 30 % of patients considered drug refractory. According to current diagnostic criteria (ICHD-3 beta), remission periods, as described above, effectively mean that the patient’s diagnostic status changes from chronic to episodic within one year. The clinical significance of this change was substantiated by an improvement in headache disability and a considerable reduction in use of acute and preventive medications.

Even though understanding of the trigeminal autonomic reflex and the involved central mechanisms has improved over the past years [14], a discussion of the possible mechanisms behind the effects observed in this study remains hypothetical. CH is the headache disorder with the strongest chronobiological traits, and it is characterized by a non-static level of activity [20]. The episodic variant has distinct periods of attacks and remissions, however, even in the state of remission, pathological processes may still be present. Likewise, despite the designation of *chronic*, CCH patients also experience fluctuations in attack frequency [20]. Therefore, the absence of manifest headache may not signal absence of pathology. In this regard, it is particularly interesting whether continued use of SPG stimulation may prolong the period of remission, or indeed, in non-remission patients, whether SPG stimulation in the attack-free state may prevent future attacks. Whether application of SPG stimulation in a preventive fashion during the remission period actually prolonged the remission period is unknown, and remains a theoretical discussion as data at present do not allow for such an analysis.

Although documentation is sparse, it is known that both episodic and chronic CH patients either spontaneously, or possibly in response to treatment, transition between phases throughout the course of their disease. A study of 189 CH patients in Italy over 10 years reports that approximately 20 % of ECH patients became chronic or experienced a combination of phases, while 47 % of CCH patients became episodic or a combination state during the same time frame [21]. A review by the same authors reported a rate of 14-39 % experiencing prolonged remission periods [22]. To underline the cyclic nature of the disorder, in a recent paper we found in fact that some patients also increase in frequency [12]. The possibility therefore exists that the observed periods of remission in this study were entirely spontaneous; however, given the different patient populations with drug refractory patients in our cohort, it seems it seems likely that the observed remission periods were induced by SPGS. Another possibility is that the remission periods were induced by the surgery associated with insertion of the microstimulator. However, the lag between surgery and remission, as well as the continued acute effectiveness of stimulation *after* the remission periods would seem to preclude this surgical effect. Additionally, no obvious pattern regarding the months during which remissions occurred can be found, suggesting that there was no relevant chronobiological influence. Therefore, it is likely that the remission periods were in fact induced by SPG neuromodulation. Considering that many of the observed remission periods were quite long, one might expect a larger percentage of patients to have reduced or stopped preventive medication. However, CH patients are often quite reluctant to reduce preventive medication during remission periods in fear of triggering a new cluster. This tendency may explain the reduction in preventive medications in only ~1/3 of the remission patients.

While a mechanism of action remains unknown, several hypotheses for how SPG stimulation may induce a preventive effect could be proposed. SPG stimulation may cause activation of sensory fibers from the second division of the trigeminal nerve (V2). V2 axons traverse the SPG and converge on second-order neurons in the trigeminal nucleus caudalis (TNC) together with V1 afferents. Alternatively, antidromic input to the SSN may produce changes in brainstem and hypothalamic circuits involved in CH attacks [23].

Major unresolved issues remain nosologic. Can a CCH patient using ongoing SPG stimulation who has been pain-free for at least one month be reclassified as ECH? Must the patient have at least two cluster periods separated by at least one month of no attacks to be classified as ECH? When CCH patients are treated with SPG stimulation or daily drug-based preventive therapy and have no attacks while receiving treatment, should the diagnosis be changed to ECH? How long must a CH patient be without any attacks before being re-classified as in complete remission? Of note, the term complete CH remission is not covered by the ICHD-3 (beta) which likely needs to be defined and adopted.

Our analysis has several limitations, primarily stemming from the fact that the preventive effect was unexpected at the time of the original protocol design which only evaluated acute effectiveness in a controlled manner where each patient served as their own control. To this end, only a brief baseline period of four weeks was required, however, this rather short baseline proved to be a limitation with regards to evaluating the remission periods, as it cannot be ruled out completely that the observed remissions are spontaneous. Individual patient histories concerning the change in subtype (ECH, CCH) were not systematically collected at baseline. However, as all patients enrolled had been diagnosed with CCH without periods of remission for at least one year, it is unlikely that these periods of remission were purely due to natural fluctuations in attack frequency. Furthermore, during the second year of the study, data were collected every three months retrospectively for the preceding month. Therefore, attack frequency data were imputed to fill the gaps of two months and were thus also subject to recall bias in patients not using a diary. Since by definition this study evaluated periods of remission lasting greater than one month, we believe the impact of this imputation to be negligible.

As the remission periods were unanticipated, provocation of attacks with alcohol or nitroglycerine was not attempted. Further, instruction on and use of SPG stimulation outside of attacks was not consistent. Thus, it is difficult to draw conclusions regarding the impact of preventive stimulation, or even the implantation procedure itself and further studies in this regard are warranted. Although patients were asked to treat each attack with SPG stimulation, we cannot rule out that few attacks were not recorded in the electronic diary in patients who chose not to treat attacks.

Finally, it is worth contrasting the effectiveness results from this 24 month follow-up open-label study with those open-label extension trials required by the US and other competent authorities for establishing safety of preventive medications. In the typical pharmacologic open label extension trials, only the responders usually remain on the medication for the duration of the study, while non-responders drop out. This selection bias is one of the reasons why open label extension trials are not used for efficacy evaluations. However, with an implanted device, all of the patients agreeing to participate were followed for 24 months, allowing for evaluation of efficacy in the entire group. The entire implanted group remained intact for the study period, thus effectiveness is a reasonable outcome assessment. Therefore, detailed trial guidelines for study design and outcome parameters in the area of neuromodulation should be developed.

## Conclusions

SPG stimulation provides not only acute effectiveness but also reduces attack frequency. In reducing the frequency, some patients experience complete attack remission, effectively converting to the episodic CH subtype. SPG stimulation’s ability to acutely treat CH attacks is maintained, providing acute relief both before and after remission. Following the remission, headache disability is reduced, and some patients can also reduce their use of medications. Long term investigations into remission periods and possible pathophysiological mechanisms are warranted, as is consideration for re-definitions of episodic CH and complete remission.

## Abbreviations

CCH, chronic cluster headache; CH, cluster headache; CPS, categorical pain scale; ECH, episodic cluster headache; HIT-6, headache impact test; LTFU, long-term follow-up; SPG, sphenopalatine ganglion; SSN, superior salivatory nucleus; TNC, trigeminal nucleus caudalis; V2, second division of the trigeminal nerve

## References

[1] The International Classification of Headache Disorders, 3rd edition (beta version) (2013). Cephalalgia 33 (9):629-808. doi: 10.1177/0333102413485658 10.1177/0333102413485658 23771276

[2] Jurgens TP, Gaul C, Lindwurm A, Dresler T, Paelecke-Habermann Y, Schmidt-Wilcke T, Lurding R, Henkel K, Leinisch E (2011). Impairment in episodic and chronic cluster headache. Cephalalgia.

[3] D'Amico D, Rigamonti A, Solari A, Leone M, Usai S, Grazzi L, Bussone G (2002). Health-related quality of life in patients with cluster headache during active periods. Cephalalgia.

[4] Fischera M, Marziniak M, Gralow I, Evers S (2008). The incidence and prevalence of cluster headache: a meta-analysis of population-based studies. Cephalalgia.

[5] Gaul C, Finken J, Biermann J, Mostardt S, Diener HC, Muller O, Wasem J, Neumann A (2011). Treatment costs and indirect costs of cluster headache: a health economics analysis. Cephalalgia.

[6] Beck E, Sieber WJ, Trejo R (2005). Management of cluster headache. Am Fam Physician.

[7] Goadsby PJ, Schoenen J, Ferrari MD, Silberstein SD, Dodick D (2006). Towards a definition of intractable headache for use in clinical practice and trials. Cephalalgia.

[8] Sluder G. The role of the sphenopalatine (or Meckel's) ganglion in nasal headaches. NY Med J. 1908;87:989-90.

[9] May A, Goadsby PJ (1999). The trigeminovascular system in humans: pathophysiologic implications for primary headache syndromes of the neural influences on the cerebral circulation. J Cereb Blood Flow Metab.

[10] Assaf AT, Hillerup S, Rostgaard J, Puche M, Blessmann M, Kohlmeier C, Pohlenz P, Klatt JC, Heiland M, Caparso A, Papay F (2016). Technical and surgical aspects of the sphenopalatine ganglion (SPG) microstimulator insertion procedure. Int J Oral Maxillofac Surg.

[11] Schoenen J, Jensen RH, Lanteri-Minet M, Lainez MJ, Gaul C, Goodman AM, Caparso A, May A (2013). Stimulation of the sphenopalatine ganglion (SPG) for cluster headache treatment. Pathway CH-1: a randomized, sham-controlled study. Cephalalgia.

[12] Jurgens TP, Barloese M, May A, Lainez MJ, Schoenen J, Gaul C, Goodman AM, Caparso A, Jensen RH (2016) Long-term effectiveness of sphenopalatine ganglion stimulation for cluster headache. Cephalalgia (in press) 10.1177/0333102416649092 PMC5405839 27165493

[13] Schytz HW, Barlose M, Guo S, Selb J, Caparso A, Jensen R, Ashina M (2013). Experimental activation of the sphenopalatine ganglion provokes cluster-like attacks in humans. Cephalalgia.

[14] Akerman S, Holland PR, Lasalandra MP, Goadsby PJ (2009). Oxygen inhibits neuronal activation in the trigeminocervical complex after stimulation of trigeminal autonomic reflex, but not during direct dural activation of trigeminal afferents. Headache.

[15] Assaf AT, Klatt JC, Blessmann M, Kohlmeier C, Friedrich RE, Pohlenz P, May A, Heiland M, Jurgens TP (2015) Value of intra- and post-operative cone beam computed tomography (CBCT) for positioning control of a sphenopalatine ganglion neurostimulator in patients with chronic cluster headache. J Craniomaxillofac Surg. doi: 10.1016/j.jcms.2014.12.017 10.1016/j.jcms.2014.12.017 25648069

[16] Jurgens TP, Schoenen J, Rostgaard J, Hillerup S, Lainez MJ, Assaf AT, May A, Jensen RH (2014). Stimulation of the sphenopalatine ganglion in intractable cluster headache: expert consensus on patient selection and standards of care. Cephalalgia.

[17] Mitsikostas DD, Edvinsson L, Jensen RH, Katsarava Z, Lampl C, Negro A, Osipova V, Paemeleire K, Siva A, Valade D, Martelletti P (2014). Refractory chronic cluster headache: a consensus statement on clinical definition from the European Headache Federation. J Headache Pain.

[18] Martelletti P, Jensen RH, Antal A, Arcioni R, Brighina F, de Tommaso M, Franzini A, Fontaine D, Heiland M, Jurgens TP, Leone M, Magis D, Paemeleire K, Palmisani S, Paulus W, May A (2013). Neuromodulation of chronic headaches: position statement from the European Headache Federation. J Headache Pain.

[19] Smelt AF, Assendelft WJ, Terwee CB, Ferrari MD, Blom JW (2014). What is a clinically relevant change on the HIT-6 questionnaire? An estimation in a primary-care population of migraine patients. Cephalalgia.

[20] Barloese M, Lund N, Petersen A, Rasmussen M, Jennum P, Jensen R (2015) Sleep and chronobiology in cluster headache. Cephalalgia. doi: 10.1177/0333102414564892 10.1177/0333102414564892 25573893

[21] Manzoni GC, Micieli G, Granella F, Tassorelli C, Zanferrari C, Cavallini A (1991). Cluster headache--course over ten years in 189 patients. Cephalalgia.

[22] Torelli P, Manzoni GC (2002). What predicts evolution from episodic to chronic cluster headache?. Curr Pain Headache Rep.

[23] Ansarinia M, Rezai A, Tepper SJ, Steiner CP, Stump J, Stanton-Hicks M, Machado A, Narouze S (2010). Electrical stimulation of sphenopalatine ganglion for acute treatment of cluster headaches. Headache.

